# Endocan: A Key Player of Cardiovascular Disease

**DOI:** 10.3389/fcvm.2021.798699

**Published:** 2022-01-05

**Authors:** Jinzhi Chen, Liping Jiang, Xiao-Hua Yu, Mi Hu, Yang-Kai Zhang, Xin Liu, Pingping He, Xinping Ouyang

**Affiliations:** ^1^Hengyang Key Laboratory of Neurodegeneration and Cognitive Impairment, Department of Physiology, Basic Medical School, Hengyang Medical College, The Neuroscience Institute, University of South China, Hengyang, China; ^2^Clinical Drug Research Center, Hunan Taihe Hospital, Changsha, China; ^3^Institute of Clinical Medicine, The Second Affiliated Hospital of Hainan Medical University, Haikou, China; ^4^Hengyang Medical School, The Affiliated Changsha Central Hospital, University of South China, Hengyang, China; ^5^School of Nursing, Hengyang Medical College, University of South China, Hengyang, China

**Keywords:** endocan, atherosclerosis, inflammation, endothelial dysfunction, CVD

## Abstract

Endothelial dysfunction is considered to be an early change in atherosclerosis. Endocan, also known as endothelial cell specific molecule-1, is a soluble proteoglycan mainly secreted by endothelial cells. Inflammatory factors such as IL-1β and TNF-α can up regulate the expression of endocan and then affect the expression of cell adhesion molecules, such as ICAM-1 and VCAM-1, which play an important role in promoting leukocyte migration and inflammatory response. Elevated plasma levels of endocan may reflect endothelial activation and dysfunction, and is considered to be a potential immuno-inflammatory marker that may be related to cardiovascular disease. In the case of hypertension, diabetes, angina pectoris and acute myocardial infarction, the increase or decrease of serum endocan levels is of great significance. Here, we reviewed the current research on endocan, and emphasis its possible clinical value as a prognostic marker of cardiovascular disease. Endocan may be a useful biomarker for the prognosis of cardiovascular disease, but more research is needed on its mechanism of action.

## Introduction

Endocan, a soluble dermatan sulfate proteoglycan, is mainly secreted by the activated endothelium and expressed by lung and kidney endothelial cells. Its secretion is controlled by pro-inflammatory cytokines. The synthesis and secretion of endocan are up-regulated by tumor necrosis factor-α (TNF-α), interleukin-1β (IL-1β), lipopolysaccharide and angiogenic factors such as vascular endothelial growth factor (VEGF), and down-regulated by interferon-γ (1, 2). It can be up-regulated by several pro-inflammatory cytokines and pro-angiogenic factors, suggesting that it may be involved in inflammatory state. The endothelial barrier composed of vascular endothelial cells is located between the flowing blood and surrounding tissues. Endothelial cells not only serve as a barrier for mechanical protection, but also the center and active part of the two major systems in the body-immune and vascular system (3). This special position enables it to regulate angiogenesis, vascular remodeling, vascular tone, tissue-fluid homeostasis, host defense and inflammation. Impaired endothelial cell integrity and function play an important role in atherosclerosis and ischemic heart. Damage to the endothelium leads to an increase in the production of reactive oxygen species (ROS), which can reduce the production of NO by increasing the concentration of calcium ions in the cytoplasm (4, 5). The increased production of ROS and the impairment of NO availability can induce and maintain the inflammatory state of the blood vessel wall. This process promotes the recruitment of white blood cells and the influx of blood lipids and lipoproteins into the subendothelial space, leading to atherosclerotic plaque formation (6). These properties illustrate its potential role as a biomarker of endothelial dysfunction and inflammation. Endocan is a new marker of endothelial cell activation and plays an important role in the adhesion of leukocytes to endothelial cells. A number of clinical studies have found that in patients with cardiovascular disease, endocan levels are significantly increased and are independently related to soluble intercellular adhesion molecule-1 (sICAM-1) and soluble vascular cell adhesion molecule-1 (sVCAM-1) levels (7–9).

Based on the above research results on the role of endothelial cells in atherosclerosis, although the exact role of endocan has not yet been determined, it is increasingly recognized as a promising target for predicting and further understanding atherosclerosis. In this review, we first described the general structure of endocan, briefly summarized its molecular biological characteristics, and then continued to introduce its latest research progress in cardiovascular disease and the specific mechanism.

## Structure and Expression of Endocan

**Figure 1:**
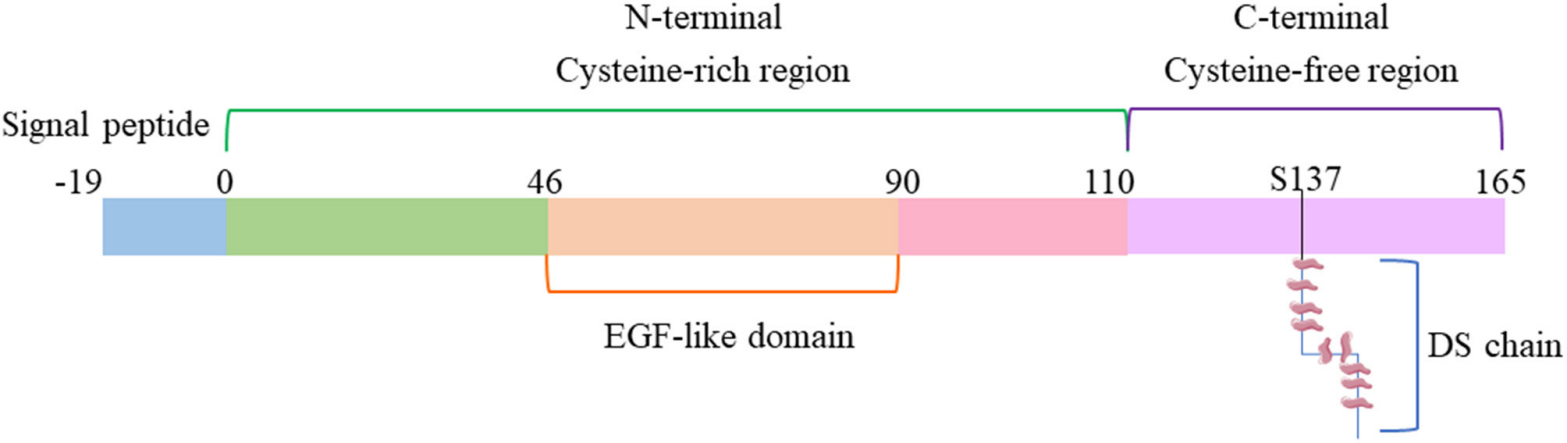
Schematic diagram of endocan structure. DS, dermatan sulfate; EGF, Epidermal Growth Factor.

Endocan is a soluble chondroitin/dermatan sulfate (DS) proteoglycan (PG). In human beings, it is encoded by the endothelial cell specific molecule-1(ESM-1) gene, and localized on the long arm of chromosome. In 1996, it was first discovered in human umbilical vein endothelial cells (10). Later in 2001, David et al. (11) proposed to change its name to “endocan” because ESM-1 is specifically secreted by endothelial cells as a proteoglycan. The molecular weight of endocan is 50 kDa. It contains a protein core, covalently linked to a glycosaminoglycan (GAG) type linear polysaccharide chain. The core protein of endocan has two different domains. The cysteine-rich domain is composed of 110 amino acids, and the other does not contain cysteine and is composed of 55 amino acids (10). The domain without cysteine is divided into three regions: endothelial growth factor-like region, phenylalanine-rich region and C-terminal region (12). During post-translational modification, the core protein is connected to the GAG chain via serine 137 (10, 11, 13) (Figure 1). Unlike most proteoglycans mainly located in the extracellular matrix (ECM) or occasionally in peripheral contact with the cell surface, endocan is mainly secreted in the blood (11, 14), the soluble form of endocan could be detected in the serum of humans.

Most of the proteoglycans containing chondroitin/DS are either ECM or related cell membranes. However, there are also a small number of PGs that are not related to ECM or cell membranes. Endocan belongs to the limited secreted proteoglycan category. The release of endocan in a soluble form increases the possibility of it spreading and regulating distal physiological processes. When compared with other PGs, endocan has a special structure. First of all, most ECM PGs are relatively large molecules containing multiple GAG chains, but endocan is smaller in size and has only a single DS chain. In comparison with the largest class of ECM PGs, such as the hyaluronan- and lectin-binding PGs, endocan does not include C-type lectin domains. Furthermore, compared with the existing DSPGs, DS chain of endocan is shorter, and the content of non-sulfate and disulfated disaccharides is higher (12, 15). This highly sulfated domain is usually associated with the high-affinity binding of growth factors. Even in the absence of alduronic acid (IdoA) residues, it can provide appropriate topological arrangements for effective binding to proteins (16). The GAG structure of proteoglycans determines their molecular interactions and properties. Although the interaction between many proteoglycans and binding proteins is mainly through GAG, there are cases where their DS chains play the same role. For example, decorin can bind to collagen through its protein core (17), and decorin can interact with another extracellular molecule tenascin-X in the matrix through its DS side chain (18). Although there is no research on this aspect of endocan, this possibility cannot be ruled out. Thus, the conformational flexibility of endocan and the binding characteristics related to its highly sulfurized domain determine its unique biological function.

Endocan is secreted by endothelial cells, but not only expressed in endothelial cells. Endocan is expressed in tissues or cells with active proliferation, such as glandular tissue, bronchial epithelium, germinal centers of lymph nodes, capillary endothelial cells, cardiomyocytes, liver cells, and neurons. On the other hand, lack of expression in relatively static tissues or cells, such as the main lung vessels, endocardium, aorta, glial cells, and vascular endothelial cells of the gastrointestinal tract.

## Regulation of Endocan Expression

Philippe et al. (10) first discovered that pro-inflammatory cytokines can promote the synthesis and secretion of endocan. The addition of TNF-α or IL-1β resulted in the upregulation of endocan expression in a time-dependent manner, while IL-4 or interferon-γ (IFN-γ) alone has no such a role. Generally, in endothelial cells, TNF-α and IFN-γ have a synergistic effect on inducing the expression of various pro-inflammatory factors. Surprisingly, in the presence of endocan, IFN-γ has no impact on the expression of endocan genes induced by TNF-α (10). This suggests that endocan may show unusual function in inflammatory response, which depends on cytokines. This result was verified in the Hsiao et al. (19) sepsis study, and he also found that VEGF-A can upregulate endocan levels. VEGF-A can specifically induce endocan transcription, while fibroblast growth factor-2 (FGF-2), platelet derived growth factor-BB (PDGF-BB), hepatocyte growth factor/scatter factor (HGF/SF) or epidermal growth factor (EGF) cannot produce this effect (20). This suggests that VEGF-A appears to be a specific inducer of endocan transcription, and the use of LY294002 to inhibit phosphoinositide 3-kinase (PI3K) will promote a 12-fold increase in endocan transcription, indicated that PI3K has an inhibitory effect (20). But inhibition of mitogen-activated protein kinase (MAPK) and c-Jun N-terminal kinase (JNK) pathways does not change the expression of endocan (21). A large number of clinical studies have found that pro-inflammatory factors IL-6 is positively correlated with endocan (22), and anti-inflammatory factor IL-10 is negatively correlated with endocan (23).

However, some studies have found different results. For instance, Zhang et al. reported that endocan can significantly reduce the levels of TNF-α, IFN-γ, IL-1β and IL-6 in a mouse model of acute lung injury (24). Endocan may relieve mitochondrial unfolded protein response and promote cell metabolic reprogramming, thereby reducing LPS-induced apoptosis. However, the intrinsic molecular mechanism of endocan down-regulating inflammatory factors and improving mitochondrial homeostasis needs further research. This may be related to the special structure of endocan. Current research has found that in different cellular environments, PGs traditionally considered to contain only HS may occasionally contain other GAGs, such as DS. For example, transforming growth factor-β(TGF-β) and calf serum (10%) can regulate the GAG chain and protein core of decorin and perlecan, TGF-β significantly increases the chondroitin sulfate (CS) chain (25). In corneal fibroblasts, fibroblast growth factor-2 has also been reported to change the expression and structure of PGs (26). Therefore, although endocan has been proven to contain DS chain, it is incorrect to believe that it is always DSPGs. Under certain conditions, its structure may change to produce different biological effects, but these require further exploration.

**Figure 2:**
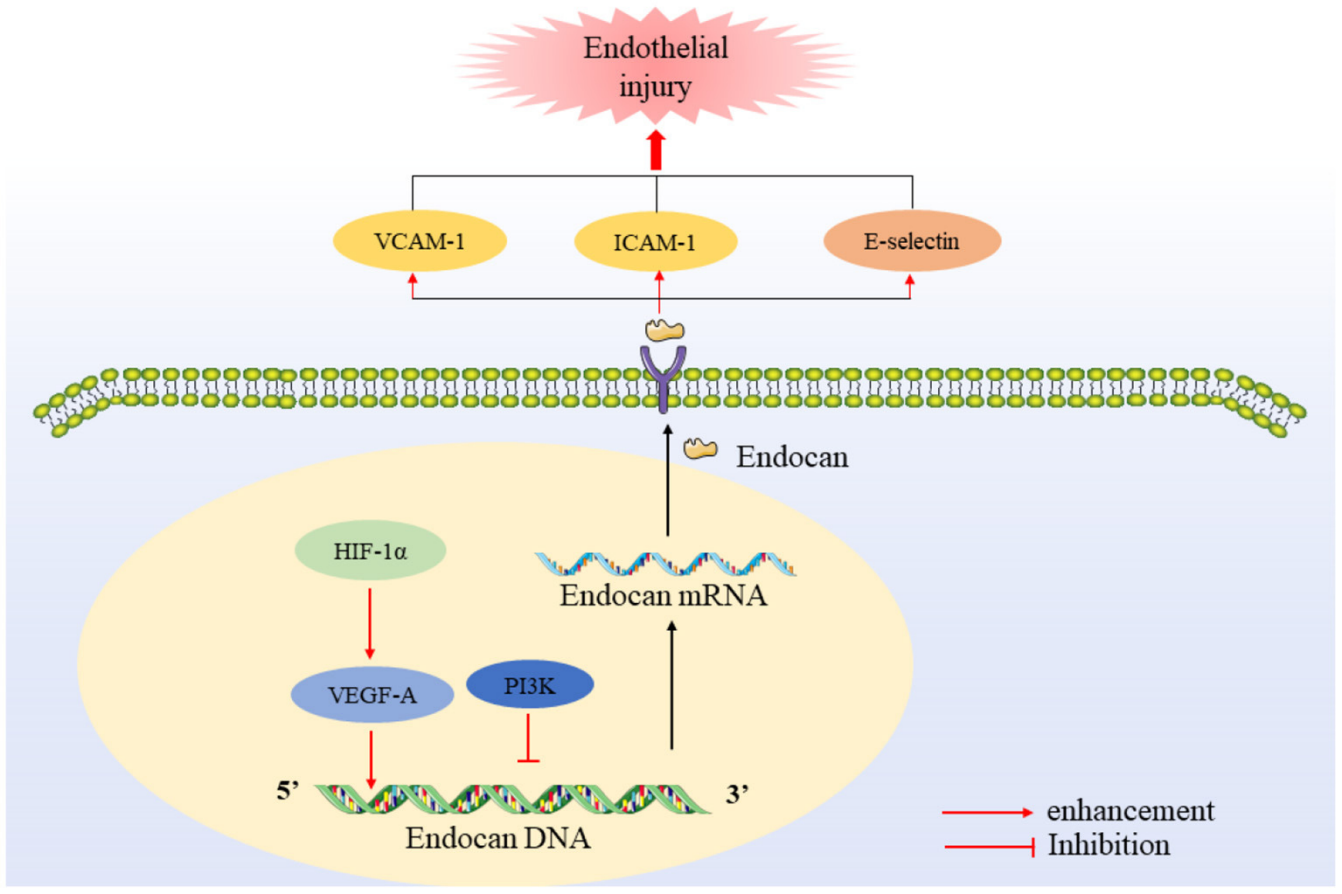
Regulation and expression of endocan. HIF-1 promotes endocan mRNA expression and transcription through VEGFA, while PI3K has the opposite effect. After being secreted, endocan promotes the up-regulation of the levels of VCAM, ICAM and E-selectin.

In addition, endocan is also a pro-inflammatory factor, because it can promote the expression of cellular adhesion molecules, such as inter-cellular adhesion molecule-1 (ICAM-1), vascular cell adhesion molecule-1 (VCAM-1) and E-selectin. ICAM-1 is an important adhesion molecule that mediates the adhesion reaction. It plays an important role in stabilizing the interaction between cells and promoting the migration of leukocytes and endothelial cells. ICAM-1 enhances the adhesion between inflammatory cells and endothelial cells, promotes the activation of endothelial cells, and makes it easier for inflammatory factors to penetrate the endothelium. ICAM-1 is most strongly expressed on proliferating vascular endothelial cells. Therefore, the adhesion between leukocytes and endothelial cells induced by ICAM-1 may be the initial event of angiogenesis. VCAM-1 is the key link between microvascular endothelial activation during inflammation and arterial endothelial cell dysfunction in atherosclerosis. VCAM-1 can selectively adhere to mononuclear leukocytes and lymphocytes, mediates the recruitment of circulating monocytes and their adhesion and migration to vascular wall, and plays a vital role in the initiation of atherosclerosis. The difference between the two is that ICAM-1 is higher than normal in all stages of atherosclerosis, while VCAM-1 only increases rapidly and significantly when the disease is initially formed, or when there is a sharp change in inflammation, such as vasospasm and plaque instability. Cell adhesion molecules ICAM-1 and VCAM-1 are adhesion receptors expressed on endothelial cells, which can up-regulate the expression of pro-inflammatory cytokines and participate in the adhesion and migration of leukocytes. Studies have found that intermittent hypoxia significantly up-regulates the expression of endocan through the hypoxia-inducible factor-1α(HIF-1α)/VEGF pathway, thereby enhancing the expression of ICAM-1 and VCAM-1 and promoting the adhesion between monocytes and endothelial cells (27) (Figure 2).

Recent studies have found that in acute respiratory distress syndrome, endocan can bind to leukocyte-binding integrin LFA-1, block its interaction with endothelial ligand ICAM-1, thereby inhibiting leukocyte recruitment (28, 29). The reason for this phenomenon may be that the binding site of endocan can be functionally regulated. Béchard et al. found that the binding of endocan to LFA-1 depends on Ca2^+^, Mg2^+^, or Mn2^+^ divalent ions, which are specific, saturable, and temperature-sensitive. The binding of endocan may depend on its single-chain glycosaminoglycan (GAG). Although the specific binding site of the lysine-rich GAG chain is not found in the LFA-1 sequence, the GAG chain of endocan is sulfated, these sulfate ions may be combined with the fixed divalent ions of LFA-1 and interfered with the binding of ICAM-1. In CD11a and CD18mAb, two known sites that block the interaction of LFA-1 with ICAM-1, no sites were found to inhibit the binding of endocan (30). However, in the presence of divalent ions, the binding capacity of endocan increased by an LFA-1mAb (HI111 clone) site. This shows that the presence of divalent ions can change the binding ability of endocan, and in different disease environments, endocan participates in inflammation and may play a completely opposite role.

## Endocan and the Cardiovascular System

The occurrence of cardiovascular diseases, such as hypertension, acute myocardial infarction, coronary heart disease, are closely related to endothelial injury. Clinical studies have found that endocan is associated with cardiovascular disease (Table 1). In the process of disease, there are different degrees of endothelial damage and vascular disease, accompanied by the up-regulation of inflammatory factors and adhesion molecules. The expression of endocan promotes the transport of inflammatory factors and the adhesion of leukocytes to endothelial cells. Therefore, endocan is currently considered to be a meaningful biomarker of cardiovascular disease.

**Table 1:** Serum concentration, specificity and sensitivity of endocan in diseases.

**Disease**	**Serum concentration**	**Control**	**Concentration (critical value)**	**Specificity (%)**	**Sensitivity (%)**	**PMID**
HT	1.18 ng/mL 1.31 ng/mL	0.73 ng/mL 0.74 ng/mL				25588082 24402320
Essential HT	34.2 ng/mL	24.1 ng/mL				30630336
Primary HT	2.03 ng/mL	1.09 ng/mL				29084442
HTwith CVD	1.47 ng/mL	0.73ng/mL				25588082
HT without CAD HT with CAD	1.31 ng/Ml 1.63 ng/mL	0.76 ng/mL				26200037
Obstructive CAD	382.7 pg/mL	268.0 pg/mL				26744512
Microvascular angina	324.3 pg/mL	268.0 pg/mL				26744512
Cirrhosis	2.6 (0.7–3.6) ng/mL					31539885
Cirrhosis and DM	4.08 ng/mL					31539885
Compensated cirrhosis	1.98 ng/mL	0.95 ng/mL				28614777
Decompensated cirrhotic	3.2 ng/ml (No infection) 6.2 ng/ml (infection)	0.95 ng/mL	≥2.05 ng/ml	85	76.1	28614777
T2D	491.8 pg/mL	228.7 pg/L				32767341
NAFLD	1.23 ng/mL	0.68 ng/mL				28922438
NAFLD	146.56 pg/mL	433.71 pg/mL	122.583 pg/mL	90	71.79	32317862
STEMI	1.6 ng/mL	2.7 ng/mL	1.7 ng/mL	73.6	76.1	27178721
SCF	2.07 ng/mL	3.71 ng/mL	2.3 ng/mL	75.2	77.2	26607436
ISR	2.56 ng/mL	1.43 ng/mL	1.625 ng/mL	78	86	32201461

*HT, Hypertension; CVD, Cardiovascular diseases; CAD, Coronary atherosclerotic heart disease; DM, diabetes; T2D, Type 2 diabetes; NAFLD, non-alcoholic fatty liver disease; STEMI, ST-segment elevation myocardial infarction; SCF, slow coronary flow; ISR, in-stent restenosis*.

### Endocan and Hypertension

Hypertension is a major cause of death and disease in the world. In the past 30 years, the number of adults with hypertension aged 30–79 has increased from 6.5 million to 12.8 billion. Endothelial dysfunction is one of the early and characteristic pathophysiological changes of hypertension. It plays an important role in the pathogenesis of cardiovascular and cerebrovascular events and target organ damage caused by essential hypertension. NO is the main factor of endothelial-dependent diastolic function. When endothelial function is impaired, NO releasse is significantly decreased and vascular ROS generation is increased, leading to arterial vasoconstriction and hypertension. Clinical data found that endocan remained independent and positively correlated with hypertension. For every increase of 1 pg/mL of endocan, the incidence of hypertension increased by 32.2% (31). In the early stages of hypertension, the endocan concentration in plasma is significantly increased, endocan levels are positively correlated with renal enzymes, norepinephrine (32), carotid intima-media thickness (cIMT) and high-sensitivity C-reactive protein (hsCRP) (33, 34), negatively correlated with leukocyte count (32). In addition, patients with higher endocan levels showed higher arterial pulse wave velocity, a sign of arterial stiffness (35). Therefore, circulating endocan levels may be a new marker of essential hypertension. Hypertension is one of the important factors leading to coronary heart disease. The content of serum endocan in patients with hypertension was independently correlated with the existence and severity of coronary heart disease (36, 37). These results suggest that endocan may be a useful biomarker for monitoring the development of coronary heart disease in hypertensive patients.

Hypertensive disorder complicating pregnancy is a common obstetric disease and the second leading cause of maternal death. Eclampsia is a kind of hypertension in pregnancy, and it is a common disease to threaten the life of pregnant women in worldwide. Pre-eclampsia will have pathological changes such as systemic vascular inflammation and endothelial dysfunction. Patients with pre-eclampsia have significantly higher endocan levels and average TNF-α concentrations. Serum endocan concentration in women with pre-eclampsia is positively correlated with systolic blood pressure, diastolic blood pressure and TNF-α. Plasma endocan level of patients with severe eclampsia is significantly higher than that of mild (38). Chew et al. found that endocan is highly expressed in fetal endothelial cells, maternal endothelial cells and decidual cells in the case of hypertension during pregnancy, and endocan expression is related to fetal low birth weight and premature delivery (39). This may be related to placental growth factor, which has a unique regulating effect on the function of trophoblast cells and endothelial cells, and can promote angiogenesis. Hentschke et al. found that there is a negative correlation between endocan and placental growth factor in both the normotensive group and the preeclampsia group. Similarly, higher levels of endocan were found in the maternal plasma of the pre-eclampsia group, and the placental endocan levels of pre-eclampsia were lower (40). In addition, it was also found that the endocan concentration in maternal and fetal plasma was positively correlated, while the endocan level in maternal/fetal plasma was negatively correlated with fetal birth weight, placental weight, and gestational age (41). On the contrary, comparing healthy pregnant women with pregnant women with pre-eclampsia, there was no difference in endocan levels between the two groups (42). Except that endocan is negatively correlated with BMI, it has no correlation with other indicators. Szpera-Gozdziewicz et al. also found that there is no significant difference in endocan levels in patients with hypertensive disorder complicating pregnancy, which indicates that endocan is not involved in the pathogenesis of hypertensive disorder complicating pregnancy (43). There may be two reasons for the differences in the research results: one is the differences in race and age of the subjects; the other is that the increase of endocan level may be directly proportional to fetal age (44), because progressive endothelial dysfunction gradually develops with the increase of fetal age. So far, few publications have been published in this field, and these findings are uncertain. Therefore, further research is needed to study this field.

### Endocan and Coronary Artery Disease

Coronary artery disease (CAD) refers to the disease caused by partial or total obstruction of coronary artery supplying blood to myocardium. Atherosclerosis is the most common cause of abnormal reduction of cardiac blood supply. A meta-analysis found that the serum endocan content of patients with coronary artery disease and slow coronary flow was higher than the control group (45). Ziaee et al. registered 340 patients with acute coronary syndromes, including ST-segment elevation myocardial infarction (STEMI), non-STEMI or unstable angina pectoris. Clinical data found that serum endocan levels are positively correlated with myocardial infarction thrombolysis risk score and major adverse cardiac events (MACE), and are independently correlated with MACE (46). In addition, the endocan level is independently related to the presence of STEMI (47). In patients with acute myocardial infarction, the level of endocan and hsCRP were also found to be significantly increased, but there was no significant correlation between endocan and hsCRP levels (48). Heart failure is the final stage of cardiovascular disease. During clinical follow-up, it was found that the average plasma endocan level in patients with chronic heart failure was 3.38 ng/mL (the normal level is 1 ng/ml). There is a correlation between the endocan level and the occurrence of heart failure (49). Endocan is an independent predictor of heart failure-related events in patients with chronic heart failure. These results indicate that endocan may be a useful predictor of coronary artery disease mortality and disease severity. Statins are the most commonly used drugs for clinical prevention and treatment of coronary heart disease. Administration of atorvastatin at 80 mg did not significantly reduce endocan levels, but 40 mg of rosuvastatin did so, which indicated that rosuvastatin may be more effective in reducing endocan levels (50). This may be related to the lipid-lowering rate of the two statins, but the specific reason is not clear.

The occurrence of coronary accessory circulation (CCC) is an adaptive response to chronic myocardial ischemia. The increase in blood flow to the side of the coronary arteries can reduce the occurrence of angina pectoris and cardiovascular events. Clinical studies have found that low endocan levels are independently associated with good CCC, low levels of hsCRP are also independently associated with good CCC (51). Current studies have found that endocan has a strong correlation with cardiovascular disease, especially the occurrence of coronary artery disease. Endocan is expected to become a useful biological indicator of coronary artery disease.

### Endocan and Atherosclerosis

Atherosclerosis (AS) is the pathophysiological basis of coronary heart disease, cerebral infarction, and peripheral vascular disease. Its features are that the affected arterial disease starts from the intima. Slow coronary flow (SCF) means that there is no obvious disease in the coronary arteries during coronary angiography, but the blood perfusion is delayed at the distal end. The phenomenon of SCF rate has recently been referred to as an atherosclerotic process, which may be caused by an increase in inflammation. SCF may cause myocardial ischemia, angina pectoris and myocardial infarction. Studies have found that endocan levels are significantly positively correlated with hsCRP, and the endocan level is independently related to the presence of SCF (52). Serum endocan levels in patients with aortic atherosclerosis stroke are significantly increased. The endocan level began to decline on the 6th day after ischemic stroke, and returned to normal at the 4th week. Higher endocan levels in serum of patients with aortic atherosclerotic stroke can help predict short-term adverse outcomes (53). At the same time, in mice lacked apolipoprotein E(ApoE)/low density lipoprotein receptors, endocan levels were found to increase in the early stages of atherosclerotic plaque development (54). These studies indicate that endocan may have been involved in the occurrence and development of atherosclerosis at an early stage.

Systemic lupus erythematosus (SLE) is a chronic inflammatory autoimmune disease, closely related to atherosclerotic diseases. SLE patients not only have an increase in the prevalence of CAD, but also have the characteristics of severe coronary artery disease and an earlier age of onset. The serum endocan concentration of SLE patients was significantly higher than that of healthy people (55). Serum endocan level are positively correlated with cIMT, body mass index (BMI) and erythrocyte sedimentation rate in patients with SLE (56). Psoriasis is a chronic inflammatory disease that may be related to atherosclerosis, myocardial infarction or stroke. The occurrence of psoriasis may be closely related to inflammation, oxidative stress, and endothelial dysfunction (57, 58). Serum TNF-α and endocan levels in patients with psoriasis were significantly increased, and consistent with previous studies of coronary heart disease, endocan levels were positively correlated with TNF-α and the average carotid intimal media thickness. And in patients with psoriasis, it was found that the serum concentration of endocan was positively correlated with the psoriasis area and severity index) score and BMI, and was negatively correlated with the age of onset of psoriasis (59). At the same time, studies have shown that endocan can be used as an early predictor of increased carotid intimal media thickness, and that elevated endocan levels may be the basis for the development of psoriasis-related cardiac manifestations (60). This finding indicates that the level of endocan is related to subclinical atherosclerosis associated with SLE and psoriasis, may be a useful biological indicator for predicting early development.

**Figure 3:**
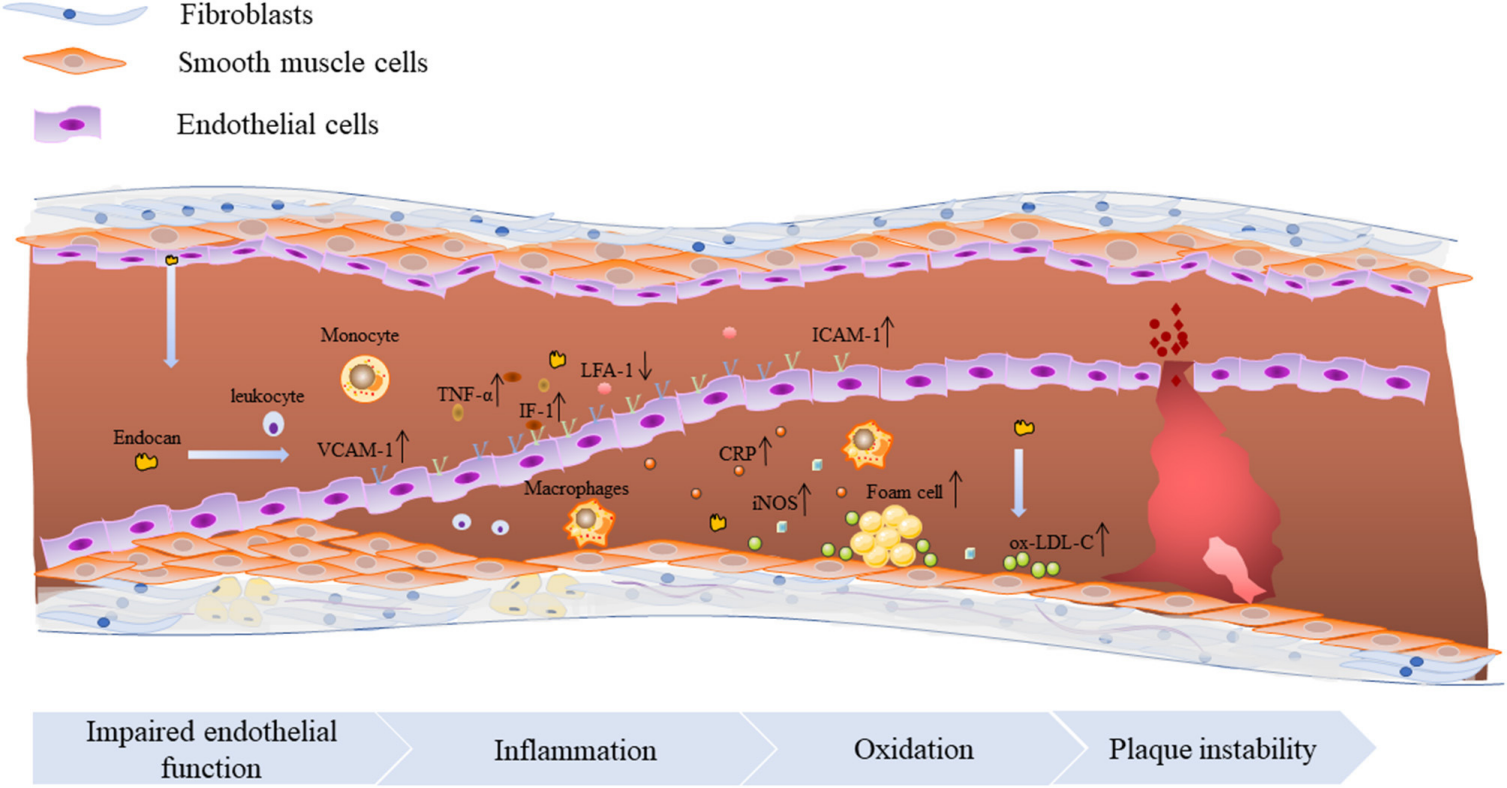
The molecular mechanism of endocan promoting atherosclerosis. Endocan promotes the occurrence and development of atherosclerosis through a variety of ways, including promoting inflammation, leukocyte proliferation and migration, oxidative stress and foam cell formation.

Chronic inflammation promotes the process of atherosclerosis in almost all stages by activating endothelial cells, producing reactive oxygen species, and accelerating the formation of foam cells and atherosclerotic plaques. At present, it is generally believed that endocan is involved in the development of atherosclerosis mainly through endothelial dysfunction. The expression of endocan increases under the stimulation of IL-1β, TNF-α and other pro-inflammatory factors, which further increases the levels of VCAM-1 and ICAM-1, thereby enhancing the adhesion between leukocytes and endothelial cells, and promoting the recruitment and migration of inflammatory cells. In addition, endocan treatment can stimulate the production of NO and ROS, and increase the expression of iNOS and CRP in RAW264.7 macrophages (5) (Figure 3). These studies prove that endocan can accelerate endothelial cell dysfunction by promoting inflammation, cell adhesion and oxidative stress.

Interestingly, compared with normal people, type 2 diabete patients show higher levels of the smallest high-density lipoprotein (HDL) particles HDL3c. There is a significant positive correlation between endocan and LDL diameter, but a negative correlation with the proportion of sdLDL particles (61).

### Endocan and Lipid Metabolism Disorder

Lipid metabolism plays a key role in maintaining the homeostasis of the systemic lipid pool. Disorders of lipid metabolism can lead to diseases such as obese, hyperlipidemia, non-alcoholic fatty liver disease (NAFLD), diabetes, and Alzheimer's disease. Both NAFLD and diabetes are independent factors for cardiovascular events. Obesity is a risk factor for atherosclerosis. These diseases have an inseparable relationship with inflammation. The increased inflammatory markers and production of free fatty acids and free radicals, unbalanced antioxidant capacity and liver cell dysfunction, are some of the potential mechanisms between obesity and cardiovascular disease (CVD). As expected, the current research has found that endocan does have an important effect in these diseases, but the results of the research are quite divergent. Nalbantoglu et al. (62) found that compared with the control group, obese children have higher endocan content. This group of people also have increased fasting insulin levels and cIMT. Consistent with previous studies, there is a significant correlation between cIMT and endocan in obese children. There is a positive correlation between endocan and almost all anthropometric data [i.e., body adiposity index (BAI), cardiometabolic index (CMI), body mass index, fat accumulation index, visceral fat index, BMI, waist circumference, hip circumference, waist-to-height ratio and waist-to-hip ratio]. Among them, non-traditional obesity index, BAI and CMI are independently related to higher serum glucose levels in adults (63). NAFLD subjects had significantly higher endocan levels than control group, and showed higher ALT and insulin levels, as well as more severe dyslipidemia (64, 65). P-selectin is a transmembrane glycoprotein expressed on the surface of platelets and ECs. Under the normal condition, P-selectin is mainly stored in platelet α particles and Weibel-Palade bodies of ECs. There was no or low expression on the surface of cell membrane. It can be rapidly expressed on the plasma membrane under the action of inflammatory injury or agonist, and plays a key role in vascular inflammation and injury. Plasma P-selectin levels are elevated in patients with hypertension (66), hyperlipidemia (67), and other atherosclerotic diseases (68). In obese subjects with NAFLD, compared with the simple obesity group and control group, the level of P-selectin decreased, and the level of circulating P-selectin was closely related to endocan (65). Endocan also seems to be associated with bacterial infection in patients with liver cirrhosis. Studies have found that liver cirrhosis patients with infection have significantly higher serum endocan, CRP, TNF-α and procalcitonin levels. In the uninfected group of liver cirrhosis, the endocan level is not correlated with other indicators, while in the infected group, the endocan level was correlated with the child-Pugh score, CRP, and TNF-α levels (69). Spontaneous bacterial peritonitis (SBP) is one of the most common bacterial infections in patients with liver cirrhosis, especially advanced liver disease. The high level of serum endocan is an independent and important risk factor for the development of SBP (70). In all patients with liver cirrhosis, when the serum endocan level is ≥2.05 ng/ml, the detection sensitivity is 76.1% and the specificity is 85% (69). The 5 and 10-year cumulative survival rates of patients with serum endocan levels <2.0 ng/mL were 97.1% and 87.4%, while those with concentrations ≥2.0 ng/mL were 85.8 and 64.4%, respectively (71). In patients with liver cancer, elevated serum endocan and VEGF levels are significantly associated with liver dysfunction and tumor progression (72). Serum endocan level may be an independent prognostic biomarker of infection and mortality in patients with liver cirrhosis and liver cancer, especially when combined with VEGF.

Although the above studies all believe that endocan levels are elevated in liver dysfunction, there are many studies that hold different opinions. A study of patients with chronic liver disease found that the serum endocan levels of NAFLD, chronic hepatitis B and chronic hepatitis C groups were significantly lower than those of the control group, there is a negative correlation between endocan levels and the inflammation stage of chronic hepatitis (73). Consistent with this result, the clinical study of Erman et al. also found that compared with healthy controls, the serum endocan level of NAFLD patients was lower, and its best cut-off point was 122.583 pg/mL, and the detection sensitivity and specificity of the cut-off point were 71.79 and 90%, respectively (74). In patients with cirrhotic cardiomyopathy, the level of endocan was also found to be significantly lower (75). Why is there such a difference in the results of studies on endocan levels in liver injury? The study by Klisic et al. gave a hint. First of all, it may be the difference in the sample size of participants between different studies. The next, they found that as a single predictor, endocan is insufficient in distinguishing steatosis/fibrosis. However, when tested with other biomarkers in the model (such as insulin, antihypertensive therapy, etc.), endocan showed good clinical accuracy (76). These results suggest that we should use biological indicators comprehensively in clinical practice, rather than relying solely on one indicator. Considering that NAFLD is an independent predictor of CVD, endocan may reflect the severity of liver injury. However, further studies are needed to explore the causal relationship between endocan levels, liver steatosis/fibrosis and CVD.

### Endocan and Diabetes

Atherosclerotic cardiovascular disease is the leading cause of death and disability in patients with Diabetes mellitus type 2 (T2DM). The risk of worsening atherosclerosis in patients with T2DM is dramatically higher than non-diabetics. Endocan is sharply overexpressed in the plasma of T2DM patients, especially in patients with low blood glucose regulation ability, the endocan level was significantly higher than the control group (77). In patients with T2DM, endocan and hsCRP and average ischemia-modified albumin (IMA) levels in patients with endothelial dysfunction were considerably higher than those in the control group. The 24-h urine protein excretion and carotid intimal media thickness levels in the T2DM group were positively correlated with hsCRP, IMA and endocan levels. Endocan and IMA are independent risk factors for endothelial dysfunction (78). This is consistent with the results of previous studies on patients with SLE. Endocan may be an inflammatory marker of T2DM (79).

Diabetic nephropathy is one of the most important complications of diabetic patients. It is suggested that patients with diabetic nephropathy have higher levels of endocan than patients with normal albuminuria (80). Arman et al. found that after 3 months of treatment according to the recommendations of the American Diabetes Association guidelines, the patients' serum calcineurin, urinary albumin to creatinine ratio (UACR) and endocan levels were clearly reduced, but only UACR and endocan independently related (77). Increased blood pressure in patients with massive proteinuria, but the serum albumin and endocan levels are low. There is a positive correlation between endocan and systolic blood pressure. UACR is negatively correlated with endocan, and positively correlated with systolic blood pressure, duration of diabetes and platelet distribution width (81). This shows that although angiogenesis occurs in the early stage of diabetic nephropathy, as the disease progresses, subsequent kidney damage may reduce the expression of endocan. Hyperglycemia can stimulate the expression of endocan and lowering blood sugar can reduce endocan level. The decrease in endocan concentration may be related to blood sugar control and lower UACR.

Lv et.al. also found that endocan levels in T2DM are positively correlated with glycosylated hemoglobin A1, fasting blood glucose and carotid intimal media thickness, which is consistent with previous research results (82). This indicates that serum endocan level may be a useful biomarker for early diagnosis of subclinical atherosclerosis in patients with T2DM. And compared with the diabetes group, the endocan and carotid intimal media thickness levels are higher in the T2DM with subclinical atherosclerosis group. In addition, compared with newly diagnosed untreated T2DM and no other disease or complications, T2DM patients with acute STEMI have significantly higher serum endocan levels (83, 84).

In addition to diabetic nephropathy, endocan is also closely related to peripheral neuropathy in patients with T2DM. The concentration of endocan in patients with diabetic peripheral neuropathy is higher than that in other diabetic patients and healthy controls. Similarly, the endocan level of diabetic patients is also higher than healthy controls. And a significant positive correlation was detected between endoglin, aperin and endocan levels in all groups (85).

Metformin is known as a common diabetes drug. Interestingly, treatment with metformin under high glucose conditions reduced endothelial cells viability, and thus up-regulates endocan expression. Compound C, a selective inhibitor of AMPK, markedly reduces endocan expression at high glucose concentrations or supplemented with metformin. Addition of metformin to compound C-treated HUVEC seems to reduce the inhibitory effect of compound C on endocan transcription (86).

## Conclusions

Endoocan is a new biomarker of endothelial dysfunction with great potential for therapeutic intervention, and a new inflammatory marker for patients with atherosclerosis. It is of great significance for predicting the progression and prognosis of CVD events. However, there are some differences in the current research results, which may be related to the selected population and sample size of the study subjects, but more likely to be due to the complexity of the endocan structure. Therefore, from the current research, endocan is not enough to be used as a biomarker alone, and should be used together with other disease biomarkers. When IL-1β and TNF-α in the patient's plasma are elevated, endocan can be detected as a supplement to disease risk assessment to more accurately predict the prognosis of the disease. Endocan also plays a key role in the occurrence and progression of systemic lupus erythematosus, psoriasis, and type 2 diabetes. The occurrence of these diseases will increase the incidence of cardiovascular events, but the mechanism by which endocan plays a role in these diseases is not clear. Therefore, further research is needed to clarify its exact role of endocan in the above processes.

## Author Contributions

JC, PH, and XO designed the writing framework. JC wrote the manuscript and drew the picture. LJ, X-HY, MH, Y-KZ, XL, PH, and XO revised and refined the manuscript. All authors have contributed.

## Funding

The authors gratefully acknowledge the financial supports from the Natural Science Foundation of China (No. 82170485), the Natural Science Foundation of Hunan Province, China (Grant No. 2019JJ40249), Key Project of Hunnan Provincial Department of Education (20A427), The Ministry of Education Industry-University Cooperation Collaborative Education Project (202002138007), and Hunan Provincial Innovation Foundation For Postgraduate (CX20200960).

## Conflict of Interest

The authors declare that the research was conducted in the absence of any commercial or financial relationships that could be construed as a potential conflict of interest.

## Publisher's Note

All claims expressed in this article are solely those of the authors and do not necessarily represent those of their affiliated organizations, or those of the publisher, the editors and the reviewers. Any product that may be evaluated in this article, or claim that may be made by its manufacturer, is not guaranteed or endorsed by the publisher.

## Abbreviations

TNF-α: tumor necrosis factor-α
IL-1β: interleukin-1β
VEGF: vascular endothelial growth factor
ROS: reactive oxygen species
sICAM-1: soluble intercellular adhesion molecule-1
sVCAM-1: soluble vascular cell adhesion molecule-1
DS: dermatan sulfate
PG: proteoglycan
ESM-1: endothelial cell specific molecule-1
GAG: glycosaminoglycan
ECM: extracellular matrix
IdoA: alduronic acid
IFN-γ: interferon-γ
FGF: fibroblast growth factor
PDGF: platelet derived growth factor
HGF: hepatocyte growth factor
SF: scatter factor
EGF: epidermal growth factor
PI3K: phosphoinositide 3-kinase
MAPK: mitogen-activated protein kinase
JNK: c-Jun N-terminal kinase
TGF-β: transforming growth factor-β
cIMT: carotid intima-media thickness
hsCRP: high-sensitivity C-reactive protein
CAD: coronary artery disease
STEMI: ST-segment elevation myocardial infarction
MACE: major adverse cardiac events
CCC: coronary accessory circulation
AS: atherosclerosis
SCF: slow coronary flow
SLE: systemic lupus erythematosus
BMI: body mass index
HDL: high-density lipoprotein
NAFLD: non-alcoholic fatty liver disease
CVD: cardiovascular disease
BAI: body adiposity index
CMI: cardiometabolic index
SBP: spontaneous bacterial peritonitis
T2DM: diabetes mellitus type 2
IMA: ischemia-modified albumin
UACR: urinary albumin to creatinine ratio
AMPK: adenosine 5′-monophosphate (AMP)-activated protein kinase.
